# Temporal Intra-Individual Variation of Immunological Biomarkers in Type 1 Diabetes Patients: Implications for Future Use in Cross-Sectional Assessment

**DOI:** 10.1371/journal.pone.0079383

**Published:** 2013-11-04

**Authors:** Ghanashyam Sarikonda, Jeremy Pettus, Sowbarnika Sachithanantham, Sonal Phatak, Jacqueline F. Miller, Lakshmi Ganesan, Ji Chae, Ronna Mallios, Steve Edelman, Bjoern Peters, Matthias von Herrath

**Affiliations:** 1 Type 1 Diabetes Center, La Jolla Institute for Allergy and Immunology, La Jolla, California, United States of America; 2 University of California San Diego, San Diego, California, United States of America; 3 Type 1 Diabetes R&D Center, Novo Nordisk Inc., Seattle, Washington, United States of America; University of Siena, Italy

## Abstract

Multiple immune parameters such as frequencies of autoreactive CD4^+^, CD8^+^ T-cells and CD4^+^CD25^+^Foxp3^+^ T-cells have been explored as biomarkers in human T1D. However, intra-individual temporal variation of these parameters has not been assessed systematically over time. We determined the variation in each of these parameters in a cohort of T1D and healthy donors (HDs), at monthly intervals for one year. Despite low intra- and inter-assay co-efficient of variation (CV), mean CVs for each of the immune parameters were 119.1% for CD4^+^ T-cell-derived IFN-γ, 50.44% for autoreactive CD8^+^ T-cells, and 31.24% for CD4^+^CD25^+^Foxp3^+^ T-cells. Further, both HDs and T1D donors had similar CVs. The variation neither correlated with BMI, age, disease duration or insulin usage, nor were there detectable cyclical patterns of variation. However, averaging results from multiple visits for an individual provided a better estimate of the CV between visits. Based on our data we predict that by averaging values from three visits a treatment effect on these parameters with a 50% effect size could be detected with the same power using 1.8–4-fold fewer patients within a trial compared to using values from a single visit. Thus, our present data contribute to a more robust, accurate endpoint design for future clinical trials in T1D and aid in the identification of truly efficacious therapies.

## Introduction

Type 1 diabetes (T1D) is an autoimmune disease in which autoreactive T-cells destroy insulin-producing beta cells in the pancreatic islets (reviewed in 1,2). This process results in overt hyperglycemia and patients ultimately rely on exogenous insulin to maintain euglycemia. Despite recent successes in clinical trials with insulin pumps [3], and continuous glucose monitoring technologies [4], the majority of patients are unable to reach the American Diabetes Association (ADA) recommended hemoglobin A1c (HbA_1c_) glycemic target of 7% or less [5]. Further, these patients are at a higher risk for developing microvascular complications [6,7]. Though insulin is a lifesaving therapy, it does not ameliorate the underlying autoimmune process, and exogenous insulin use has major drawbacks including weight gain [8] and risk of hypoglycemia. Furthermore, while epidemiological studies have shown that incidence of T1D is rising rapidly [9-11], T1D patients remain without a therapeutic agent that can alter the underlying disease process. 

Preservation of C-peptide a surrogate marker of beta-cell preservation is the primary biomarker available for determining therapeutic efficacy in T1D subjects [12]. However, while C-peptide provides information about islet function, it does not provide insight into the underlying immune process. Thus, immunological biomarkers [13] that could reliably demonstrate a therapeutic effect on the immune system can help identify patients that would respond to a therapy, and in doing so, reduce the time required to run these clinical trials and be more cost effective. Towards this goal, several immune parameters, including CD4^+^CD25^+^Foxp3^+^ T-cells [14-16] and autoreactive CD4^+^ [17] and CD8^+^ T-cells [18] have been explored as possible biomarkers. Most of these parameters exhibit significant differences between healthy subjects and patients with T1D, potentially serving as diagnostic biomarker. However, the ability of these immune parameters to serve as biomarkers of clinical efficacy within T1D patients is largely unknown. Further, the natural (biological, inherent) variability over time of the proposed immunological biomarkers in a T1D subject not undergoing immune intervention is undetermined. 

Few prior publications have analyzed variation in some of these parameters separately e.g., changes in CD4^+^CD25^+^Foxp3^+^ T-cell frequencies were quantified and found to be variable [19], while variation in cytokine production by CD4^+^ T-cells [17] and autoreactive CD8^+^ T-cells [18] was analyzed but not quantified. However, no study has precisely quantified the variation of each of these immunological parameters in the same subject cohort. The degree of intra-individual variation must be elucidated to help determine if a change seen with a given treatment represents a true therapeutic effect versus normal biological variability. In doing so, it will also allow us to have enough statistical power in future trials to observe a true effect.

We therefore set out to investigate, in a longitudinal fashion, how specific immune parameters varied over time in patients with T1D in comparison to healthy donors (HD). We followed all donors over a one-year period with monthly blood draws. At each visit, we determined CD4^+^CD25^+^Foxp3^+^ T-cell frequencies, IFN-γ cytokine production by autoreactive CD4^+^ T-cells in response to diabetes-associated islet epitopes, and frequencies of autoreactive CD8^+^ T-cells against HLA-A2-restricted antigenic epitopes. Data was then analyzed to determine overall longitudinal variation in each of these parameters, the effect of such variation on patient numbers required to see significant changes, and associations between immune and clinical parameters.

## Research Design and Methods

### Ethics Statement

University of California San Diego Institutional Review Board (UCSD IRB) and La Jolla Institute for Allergy and Immunology (LIAI) IRB approved the study protocol and informed consent procedure. Written informed consents were obtained by clinical investigators.

### Subject recruitment, study design and scheduled blood draws

We enrolled donors clinically defined as having T1D (T1D, n=33), along with healthy donors (HDs n=10) as controls. Diabetic donors were recruited at the Veterans Affairs hospital in San Diego, La Jolla Institute for Allergy and Immunology, and at Taking Control of Your Diabetes (TCOYD) educational conferences. Informed consent, study identification numbers, clinical case histories, and other information were collected and recorded by clinical investigators. HDs were recruited from a normal blood donor program, through an umbrella protocol, at LIAI and did not require separate consent forms. To maintain the anonymity of HDs, only the age and sex of the donors were recorded as biometric information. Use of glucocorticoids at the time of study served as exclusion criteria. Each subject had ~40 mL blood drawn once every month for 12 months. Blood was drawn once every week on Monday and Tuesday, in the mornings and afternoons, and both these blood draws were non-fasting blood collections. 

### PBMC Isolation

Fresh blood was collected in heparin-coated vacutainer tubes (BD Biosciences, San Diego, CA) at the two collection sites. When collected at VA hospital, it was transported immediately to LIAI for further processing. Peripheral blood was processed within four hours of collection. Peripheral blood mononuclear cells (PBMCs) were isolated using a standard Ficoll-Paque Plus (Sigma) gradient as described previously [20]. PBMCs were adjusted to a concentration of 8 × 10^6^/mL in serum-free AIM-V medium (Invitrogen Life Sciences, San Diego, CA). They were either used fresh for FACS and ELISpot or cryopreserved in 50% AIM-V media, 40% FCS, 10% DMSO and stored in liquid nitrogen until analyzed.

### HLA-typing

For HLA-A2 typing, donors were first rapidly screened using an anti-HLA-A2 antibody (eBioscience, San Diego, CA) and only those who were positive were further typed using genomic DNA. All enrolled donors had HLA-DR typing performed. HLA-A2 and HLA-DR typing from genomic DNA was performed according to standard methods [21]. 

### FACS

Multicolor flow cytometry for the analysis of various cell populations was performed as described before [22]. Briefly, 2 × 10^5^ - 1 × 10^6^ PBMCs were stained first with antibodies against CD4, CD8, CD25 and CD127, for 20 minutes on ice in FACS buffer containing 5% FCS. Intracellular staining for Foxp3 was performed using the Foxp3 staining kit (eBioscience, San Diego, CA) according to the manufacturer’s protocol. All antibodies were obtained from eBioscience. Sample data was acquired on LSR-II (BD Biosciences, San Diego, CA) and analyzed using FlowJo software (Tree Star Inc, Ashland, OR).

### ELISpot

The ELISpot assay was performed as previously described [20] with slight modifications. To reduce non-specific background spot formation (artifact) caused by components present in human AB serum, we used serum-free AIM-V [23] (Invitrogen, Carlsbad, CA) medium throughout the ELISpot assay. DR4-restricted epitopes IA-2 (752–775) and proinsulin (C19-A3) as well as the DR3-restricted GAD 65 epitope (335–352) were used to stimulate PBMCs [20]. A pool of immunogenic viral peptides from cytomegalovirus, Epstein-Barr virus, and influenza (CEF) was used as a positive control (Mabtech Inc, Mariemont, OH). Development of interferon-gamma (IFN-γ) spots was performed using U-Cytech antibodies and reagents. High purity peptides (>95%) were obtained from Genscript and stored in small aliquots at -80 °C. Briefly, 2 × 10^6^ lymphocytes were incubated with the above epitopes (10 μg/mL) in AIM-V medium for 48 hrs. Non-adherent cells were harvested, adjusted to a concentration of 2 × 10^5^ cells per well, and plated in triplicates on maxisorp ELISA plates (Nalgene Nunc, Rochester, NY) pre-coated with capture antibody. Negative controls (cells incubated with only AIM-V medium) were also included. Cells were lysed with dH_2_O 24-hrs later, washed, and the contents were incubated with a biotinylated detection antibody. Spots were then developed using the manufacturer's reagents and protocols. Spots above 65-μM in size were counted as cytokine spots using computer-assisted image analysis [21] (KS-ELISPOT reader; Zeiss, Munich, Germany). All epitopes were run in triplicate and the positive results were averaged after subtracting background responses. Absent responses or responses not above background were not included in the analysis. 

### HLA-A2-monomer production

Peptides (>95% purity, Genscript) from HLA-A2-restricted islet antigens used for loading into HLA-A2 have been described before [18]; insulin (B-chain, aa 10–18), IA-2 (aa 797–805), IGRP (aa 265–273), PPI (aa 15–23), GAD65 (aa 114–123), and ppIAPP (aa 5–13). Peptide-loaded HLA-A*0201 monomers were produced by the NIH Tetramer Core Facility at Emory University, Atlanta, GA, according to standard protocols, http://tetramer.yerkes.emory.edu/client/protocols. 

### Qdot labeling of peptide-HLA class I monomers

Qdot labeling of HLA-A2-peptide monomers was performed as described previously [18]. Briefly, multimeric HLA-A2-peptide complexes were produced by addition of streptavidin-conjugated quantum-dots (Qdots; Invitrogen, San Diego, CA) to achieve a 1:20 streptavidin-Qdot:biotinylated pHLA ratio. The Qdots used were Qdot-585, -605, -655, -705, and -800. Each HLA-A2-monomer was labeled with two different Qdots such that each monomer had its own unique Qdot combination (Table S1). 

### Cell staining with Qdot-labeled multimeric complexes

Samples from HLA-A2–positive donors were stained with a mixture containing six diabetes-associated HLA-A2-restricted epitopes, and a mix of viral antigens (Table S1). For each donor, samples from all available visits were stained simultaneously with Qdot-HLA-A2-multimers as described previously [18]. Briefly, 2 × 10^6^ thawed PBMC were stained simultaneously with all Qdot-labeled multimers (0.1 μg each multimer) in 60 μL PBS supplemented with 0.5% BSA and incubated for 15 min at 37 °C. Subsequently, PBMC were stained with Alexa Flour-700-conjugated anti-CD8 and FITC-labeled anti-CD4, -CD14, -CD20, -CD40, and -CD16 antibodies (eBiosciences, San Diego, CA) for 30 min at 4 °C. After washing twice, cells were resuspended in PBS with 0.5% BSA containing 7-AAD (eBioscience, San Diego, CA, USA). 

### Data acquisition and analysis

Sample data were acquired using an LSRII (BD Biosciences, San Diego, CA) instrument with the following filter settings for detection of Qdots. For the 488-nm laser: Qdot 655, 635LP, and 655/20. For the 405-nm laser: QD800, 770LP, 800/30; QD705, 685LP, 710/40; QD605, 595LP, 605/20 and QD585, blank, and 585/22. Approximately 300,000 lymphocytes were recorded for each sample. To identify antigenic epitope-specific CD8^+^ T-cells, the gating strategy used was: (i) selection of live (AAD-negative) single-cell lymphocytes (FSC-H^lo^, SSC-H^lo^); (ii) selection of CD8^+^ and 'dump-channel' FITC (CD4, CD14, CD16, CD19, and CD40)-negative cells; and (iii) selection of CD8^+^ T-cells positive for two HLA-multimer channels and negative for all other HLA-multimer channels [18].

### Statistical analysis

All data were analyzed using GraphPad Prism 5 software. Statistical analyses for differences between T1D and healthy donors were tested using Mann-Whitney test assuming non-parametric distribution of the data. Correlation analyses were performed using Spearman’s rho with two-tailed significance test. All statistical analyses were performed using SPSS software (PASW Statistics 18). Power analysis and sample size calculations were performed using PASS v12 software (NCSS statistical software, Kaysville, UT).

## Results

### Donor characteristics

We recruited 33 donors with T1D and 10 HDs as controls. A summary of participant characteristics is shown in Table 1. The mean age of HDs and T1D subjects differed significantly (31.3 and 46.1 years of age, respectively). A majority of the HD cohort was female and younger; in contrast, the T1D cohort contained a similar number of males and females. Additionally, 19 out of 33 (58%) and 11 of 33 (35%) T1D donors were HLA-DR4^+^ or HLA-DR3^+^, respectively. Eight of HLA-DR3^+^ T1D donors were also HLA-DR4^+^. Among HDs, only one subject was positive for HLA-DR3 and one for HLA-DR4; further, the DR4 allele was protective (i.e., DRB1*0403). Detailed subject profiles with age, duration of disease, HLA phenotypes, and autoantibody status are shown in Table S2. 

**Table 1:** Participant information is shown including male to female ratio, age, and disease duration.

	**HD**	**T1D**
**Number of Males (%)**	1 (10)	18 (55)
**Age (Yrs)**	31.3 ± 5.27	46.06 ± 15.91
**Disease duration (Yrs)**	N/A	18.55 ± 14.93
**BMI**	N/A	25.39 ± 3.04
**Insulin dose**	N/A	40.48 ± 16.6
**TDD Insulin/Kg**	N/A	0.525 ± 0.22
**HbA1c**	N/A	7.039 ± 1.22
DRB*04:01^+^ - Number (%)	0 (0)	19 (58)
DRB*03:01^+^ - Number (%)	1 (14)	11 (36)

Mean ± SD values are shown.

The T1D cohort contained a heterogeneous distribution of male and female donors and age, but a majority of controls were female and were skewed towards a younger age. T1D donors had varying disease duration. A majority of T1D donors were HLA-DR4^+^ and some T1D donors were DR3^+^; however, only one donor each of the HD cohort was DR3^+^ or DR4^+^. The DR4 allele in HD was HLA-DRB1* 04:03. N/A represents not available (BMI and HbA_1c_ for HD) or not applicable (Duration and Insulin usage (total daily dose of insulin, TDD) for HD).

### Intra-Individual Temporal Variation in IFN-γ Production by CD4^+^ T-Cells in T1D Donors

Extensive validation of the ELISpot assay using CEF peptide pool showed the performance of the assay itself was quite reproducible, with an intra-assay co-efficient of variation (CV) of 7.3% and inter-assay CV of 13% (Figure S1). We then determined IFN-γ production in antigen-stimulated CD4^+^ T-cells using two DR4-restricted epitopes (Proinsulin, PI_C19-A3_ & IA-2_752-775_) and a DR3-restricted epitope (GAD65_335–352_), at each visit using fresh PBMCs. At each visit, we assessed the data both in terms of total net antigen-specific spot numbers (spots with antigen stimulation – spots in media only) as well as stimulation indices (SI, spots with antigen stimulation ÷ spots in media only).

**Figure 1:**
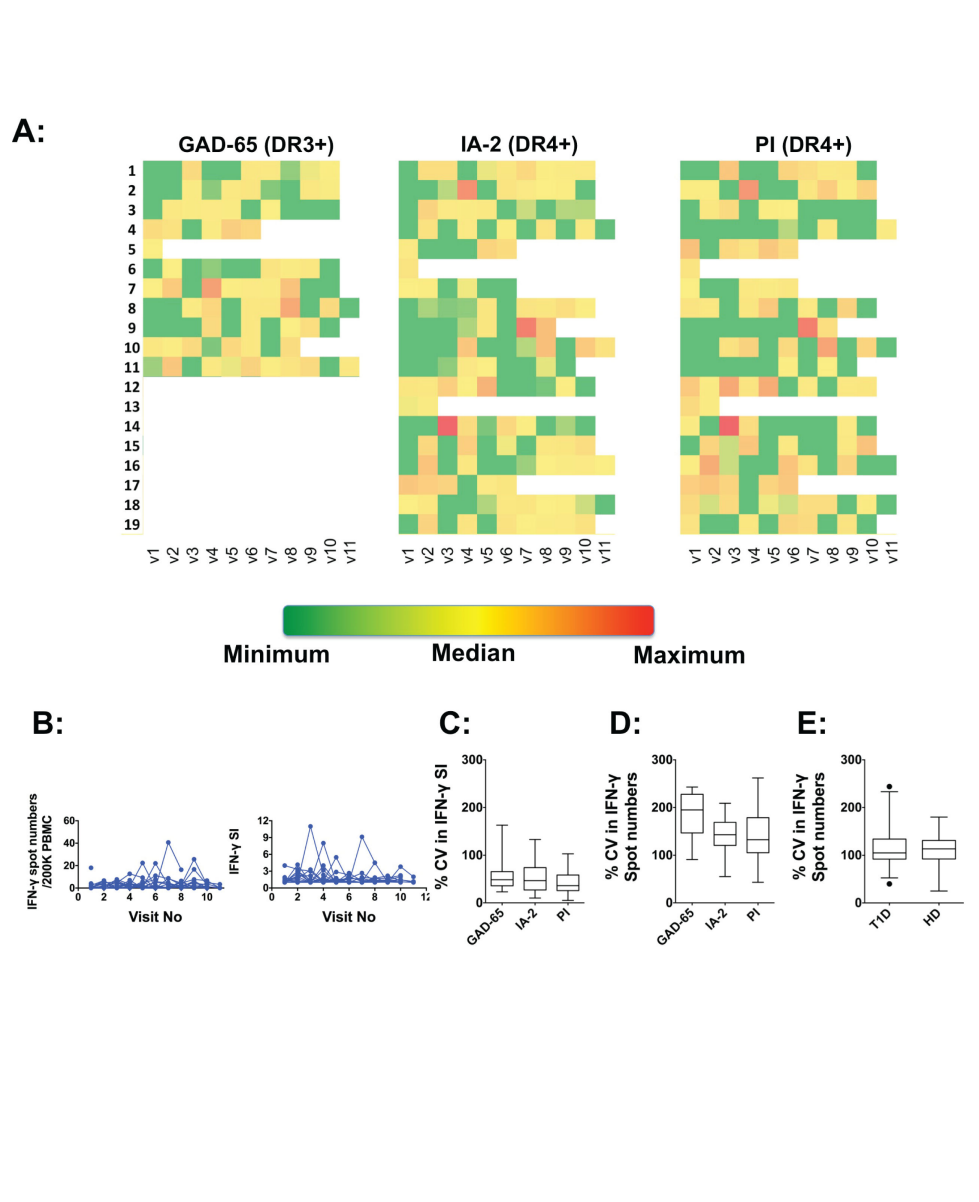
Longitudinal variation in IFN-γ production in T1D donors. At each visit, IFN-γ production against DR4-restricted epitopes of IA-2 (752–775), proinsulin (C19-A3) and a DR3-restricted epitope of GAD65 (335–352), or without stimulation (media only, no antigen) were determined. Each subject's responses to each of the three epitopes at each visit were determined as net spots (spots with antigen – spots in media) or as stimulation index (SI, spots with antigen ÷ spots in media). **A**: Data were analyzed by restricting to DR3^+^ T1D donors (GAD-65 specific response) or by restricting to DR4^+^ donors (PI- and IA-2-specific responses). SI values observed at each visit were plotted as a heat map showing minimum, median, and maximum values calculated based on values for all donors and all visits for each antigen. Each individual donor is shown on the Y-axis while each independent visit is shown on the X-axis. **B**: Longitudinal variations in net spots per 2 × 10^5^ PBMC (left panel) and SIs are shown for DR4^+^ donors and IA-2 antigen-specific responses. Each line represents an individual donor. **C**: Population mean and 95% CI of longitudinal variation in SIs assessed as CV (standard deviation ÷ mean) is shown for each antigen, in DR3^+^ (GAD-65) or DR4^+^ (IA-2 and PI) donors. **D**: Population mean and 95% confidence intervals of longitudinal variation (CV) in net spot numbers are shown for each antigen, in only DR3^+^ (GAD-65) or DR4^+^ (IA-2 and PI) donors. **E**: All T1D or control (HD) donors, irrespective of DR3- or DR4-status were included. Net spot numbers per 2 × 10^5^ PBMC for all three antigens (GAD-65, IA-2 and PI) were pooled. Population mean and 95% CI of longitudinal variation (CV) in net spot numbers are shown for all donors. For statistical analysis, non-parametric Mann-Whitney test was used to determine significance.

In Figure 1A, SI data from all available visits for each DR3^+^ (GAD-65 specific responses) or DR4^+^ (IA-2 and PI specific responses) T1D donor is shown as a heat map, with the minimum, median, and maximum values determined based on all donors. For each donor, over all visits, there were fluctuations noted in SI values on a per antigen basis (Figure 1A) for all three antigens. We also found similar fluctuations in total net spot numbers determined per each antigen. A side-by-side analysis of fluctuations in SI values and net spot numbers against IA-2 antigenic epitope is shown only for DR4^+^ T1D donors in Figure 1B (data not shown for other antigens). There was no detectable cyclical pattern to these fluctuations that emerged from assessing SIs or net spot numbers for any of the antigens.

We then quantified the observed variation in either spot numbers or SIs over all visits, as a standardized measure, i.e., co-efficient of variation, CV. We also determined the mean and 95% confidence intervals (CI) of the CV between all donors assessed for a particular antigen (only DR3^+^ for GAD-65 or only DR4^+^ for IA-2 and PI epitopes). There was significant variation in both SIs (Figure 1C) and net spot numbers (Figure 1D) regardless of antigen. While there were no apparent differences in the CV in SIs between the different antigens (Figure 1C), there were larger CVs in net spots produced in response to GAD-65 stimulation compared to IA-2 or PI stimulation (Figure 1D). 

Next, we wanted to determine whether these variations are restricted to T1D donors only or do we observe similar variations in HDs. However, our HD cohort did not have the appropriate DR3 or DR4 alleles for presentation of these antigenic epitopes. Thus, we included all T1D donors and HDs, and pooled their responses against all three epitopes, irrespective of their DR3/4-status as we and others have previously noted some HLA discordant CD4^+^ T-cell IFN-γ production [17,20]. Interestingly, both T1D and HD cohorts exhibited similar mean CV and 95% CI for IFN-γ production (Figure 1E). This analysis, despite its limitations, suggests that the longitudinal variation seen in IFN-γ production is inherent to the donor. 

### CD4^+^CD25^+^Foxp3^+^ T-cell frequencies exhibit significant temporal variation in both T1D and control donors

**Figure 2:**
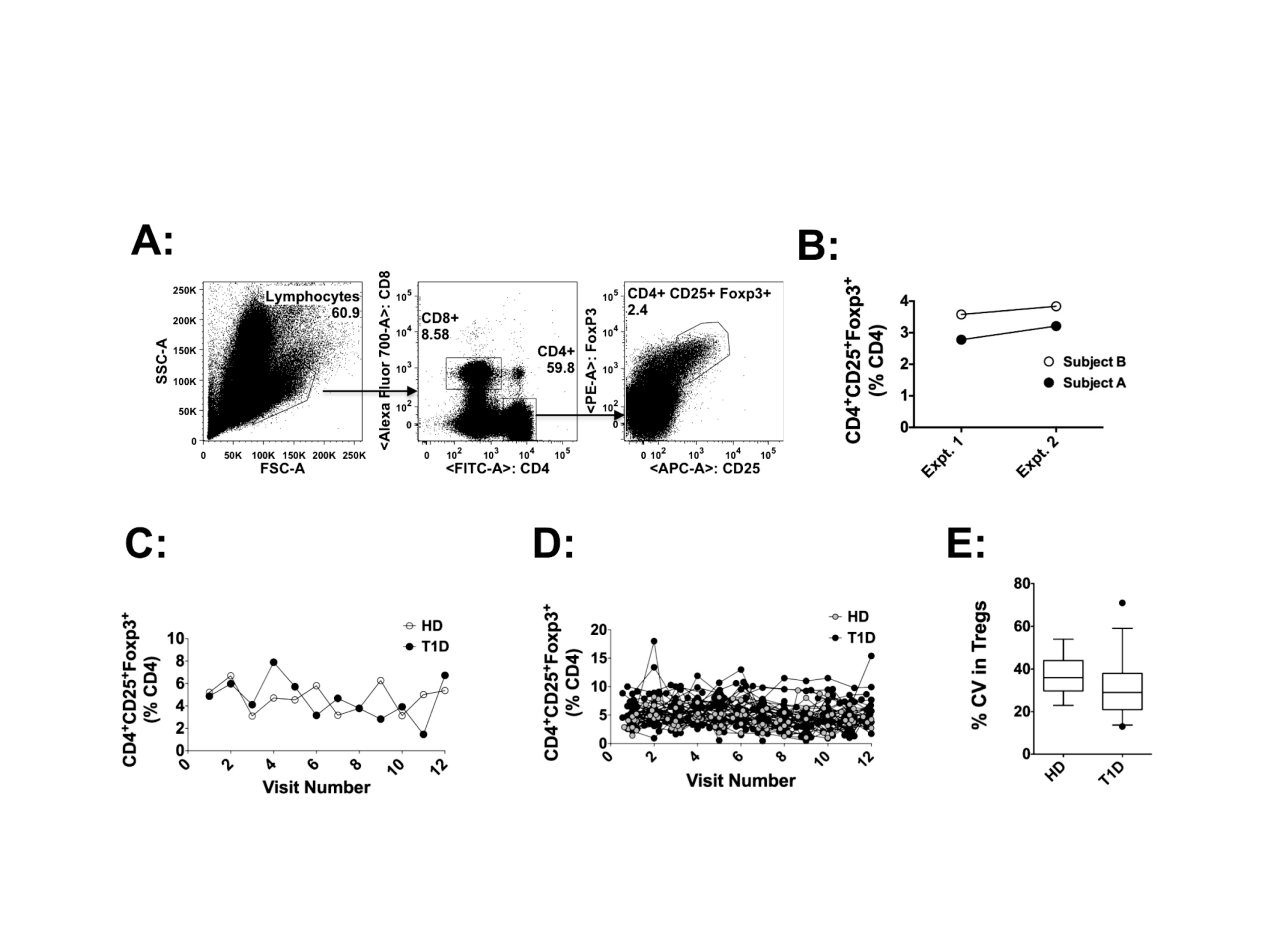
CD4^+^CD25^+^Foxp3^+^ T-cell frequencies show similar longitudinal variation between HD or T1D donors. **A**: Gating strategy used to identify CD4^+^CD25^+^Foxp3^+^ T-cells, from a representative T1D donor is shown. Frequencies of CD4^+^CD25^+^Foxp3^+^ cells (shown in FACS plots) were determined as a subset of CD4^+^ cells. **B**: Frequencies of CD4^+^CD25^+^Foxp3^+^ assessed once in the morning and once in the afternoon of the same day, in the same T1D subject, shows similar frequencies. **C**: The frequencies of CD4^+^CD25^+^Foxp3^+^ T-cell frequencies detected at each visit in one representative subject from each cohort (T1D n=33, HD n = 10) are shown. **D**: CD4^+^CD25^+^Foxp3^+^ T-cell frequencies in all donors from each cohort at each visit are shown. Each connected line represents an individual donor. **E**: Population mean and 95% CI of longitudinal variation (CV) in frequencies of CD4^+^CD25^+^Foxp3^+^ T-cells are shown for each donor cohort.

Tregs are identified by multiple phenotypic definitions, with the most widely accepted characterization being CD4^+^CD25^+^Foxp3^+^ and a CD127^lo^ staining profile. The CD4^+^CD25^+^Foxp3^+^ T-cell population is enriched for Tregs. We assessed frequencies of circulating CD4^+^CD25^+^Foxp3^+^ T-cells (Figure 2A), with CD127 co-staining in fresh peripheral blood at each of the 12 visits. A significant majority of the CD4^+^CD25^+^Foxp3^+^ cells were also CD127^lo^ (>90%, data not shown). Sampling of the same patient twice on the same day showed only minor changes in CD4^+^CD25^+^Foxp3^+^ frequencies (Figure 2B). However, between visits separated by a month, we found fluctuations in CD4^+^CD25^+^Foxp3^+^ frequencies, with some donors showing minor while some others showing major variations. Representative data of the fluctuations from one donor each in T1D and control cohort is shown in Figure 2C. Fluctuations in CD4^+^CD25^+^Foxp3^+^ T-cell frequencies among all donors in each cohort is shown in Figure 2D. A direct comparison of the CV for CD4^+^CD25^+^Foxp3^+^ T-cell numbers between HD and T1D donors revealed no major differences (Figure 2E); the mean CV and 95% CI between the two cohorts were comparable. This lack of difference between the two groups suggests that the longitudinal variation did not associate with the health status of the subject.

### Temporal variations in Ag-specific CD8^+^ T-cell numbers

We used the novel HLA-A2-Qdot-multimer technology described earlier [18] to determine the frequencies of CD8^+^ T-cells specific for six different antigenic epitopes (Table S1). In our cohort, ~50% of T1D donors (18/33) and 40% of controls (4/10) were HLA-A2^+^ (Table S2). The gating strategy used to identify antigen-specific CD8^+^ T-cells with different epitope specificities and the reproducibility of the assay is shown in Figure S2.

**Figure 3:**
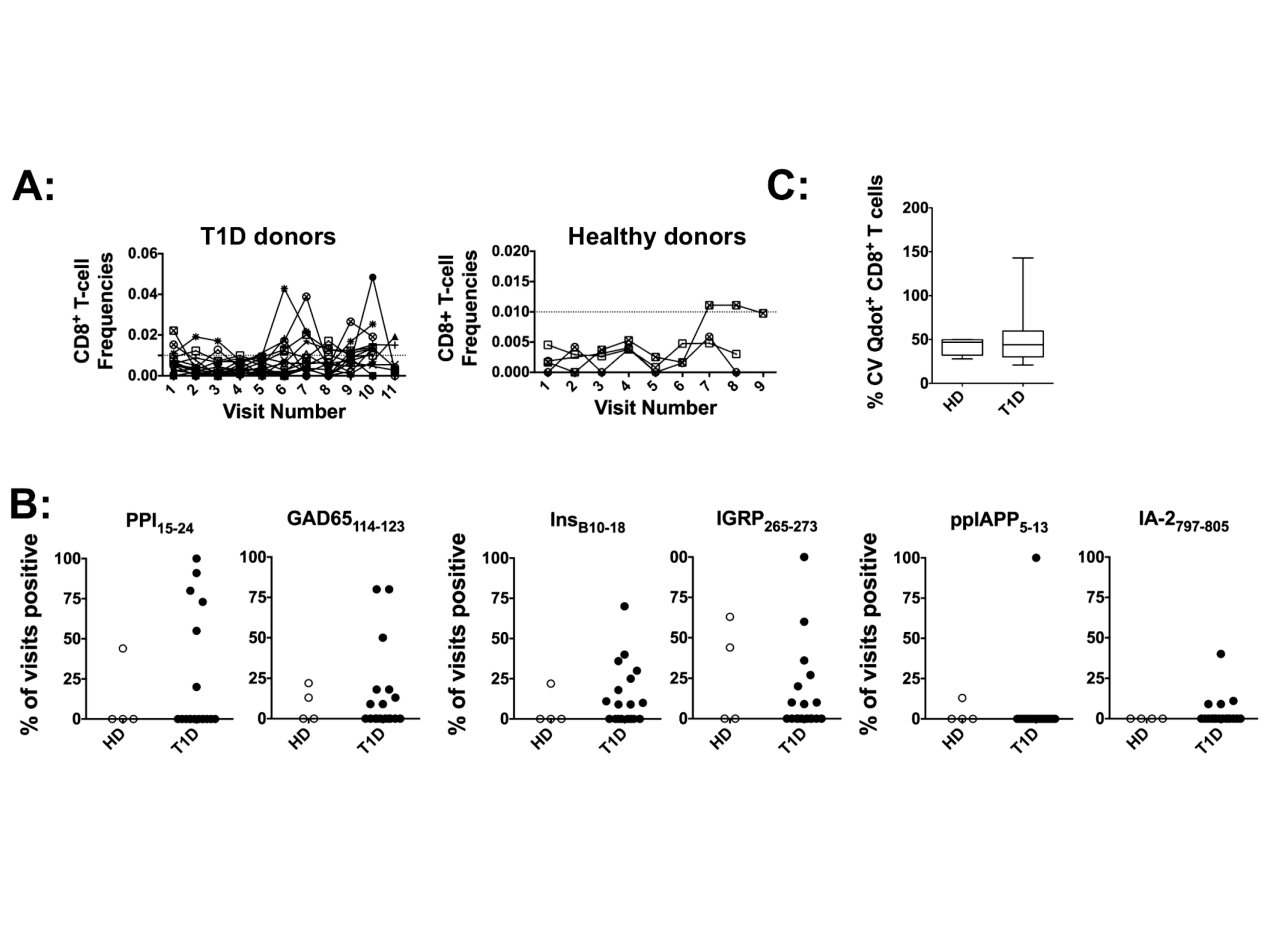
Longitudinal variation in multiple antigenic epitope specific CD8^+^ T-cells. The frequencies of CD8^+^ T-cells recognizing the diabetes-associated antigenic epitopes of insulin B_10–18_, PPI_15–24_, GAD65_114–123_, IA-2_797–805_, IGRP_265–273_, and ppIAPP_5–13_ bound to HLA-A2 were determined using flow cytometry in HLA-A2^+^ T1D (n=18), and controls (n=4). **A**: Absolute frequencies of insulin B_10–18_ epitope-specific CD8^+^ T-cells detected in each donor, over all available visits, are shown for T1D (left panel) and control (right panel) donors. Each connected line represents an individual donor. **B**: At each visit, donors were marked positive if the frequencies were above the threshold (0.01% for all except IGRP, which was 0.005%). Percentage of total visits for each donor that were positive for each antigenic epitope is shown. Each dot represents an individual donor. **C**: Absolute frequencies of all islet antigen epitope-specific CD8^+^ T-cells were pooled at each visit for each donor. Population mean and 95% CI of longitudinal variation (CV) in absolute frequencies are shown for all donors. Statistical analysis performed using the Mann-Whitney test found no significant differences between the cohorts.

We found that the frequencies of CD8^+^ T-cells specific for each epitope also exhibit fluctuations over time in both T1D and HDs. A representative analysis of InsB_10-18_-specific CD8^+^ T-cells at each visit for the T1D cohort (Figure 3A, left panel) and the HD cohort (Figure 3A, right panel) is shown, with other epitope-specific T-cells exhibiting similar fluctuations (data not shown). The frequencies of CD8^+^ T-cells detected in the HD cohort are lower than T1D donors (note the difference in y-axis scale between left and right panels of Figure 3A). Next, at each visit, when the frequencies of epitope-specific T-cells were above the threshold (0.01% of CD8^+^ T-cells for all epitopes, except for IGRP, which was 0.005%), donors were counted as positive for that visit. Using this criterion of positivity, we determined the percentage of visits in which the donors had detectable islet antigen-specific CD8^+^ T-cells. We did not find any epitope-specific CD8^+^ T-cell that was reliably detected in all visits for a majority of the donors (Figure 3B). Among donors that had detectable antigen-specific T-cells, different visits were positive for different antigen specificities. PPI- and GAD65- specific T-cells were detected with more regularity compared to insulin- or IGRP-specific T-cells while ppIAPP- or IA-2-specific T-cells were detectable for the least number of visits (Figure 3B).

Finally, as with the analysis of IFN-γ cytokine production in Figure 2E, we pooled absolute frequencies of CD8^+^ T-cells against all epitopes at each visit (only for HLA-A2^+^ donors), and determined the CV over all visits. Interestingly, similar to the CV for CD4^+^CD25^+^Foxp3^+^ T-cell numbers, frequencies of CD8^+^ T-cells also exhibited temporal variation in both T1D and HD cohorts (Figure 3C). Further, there were no significant differences in the mean or the 95% CI of CV between the HD and T1D cohorts. 

### Immunological parameters display significant longitudinal variation

**Figure 4:**
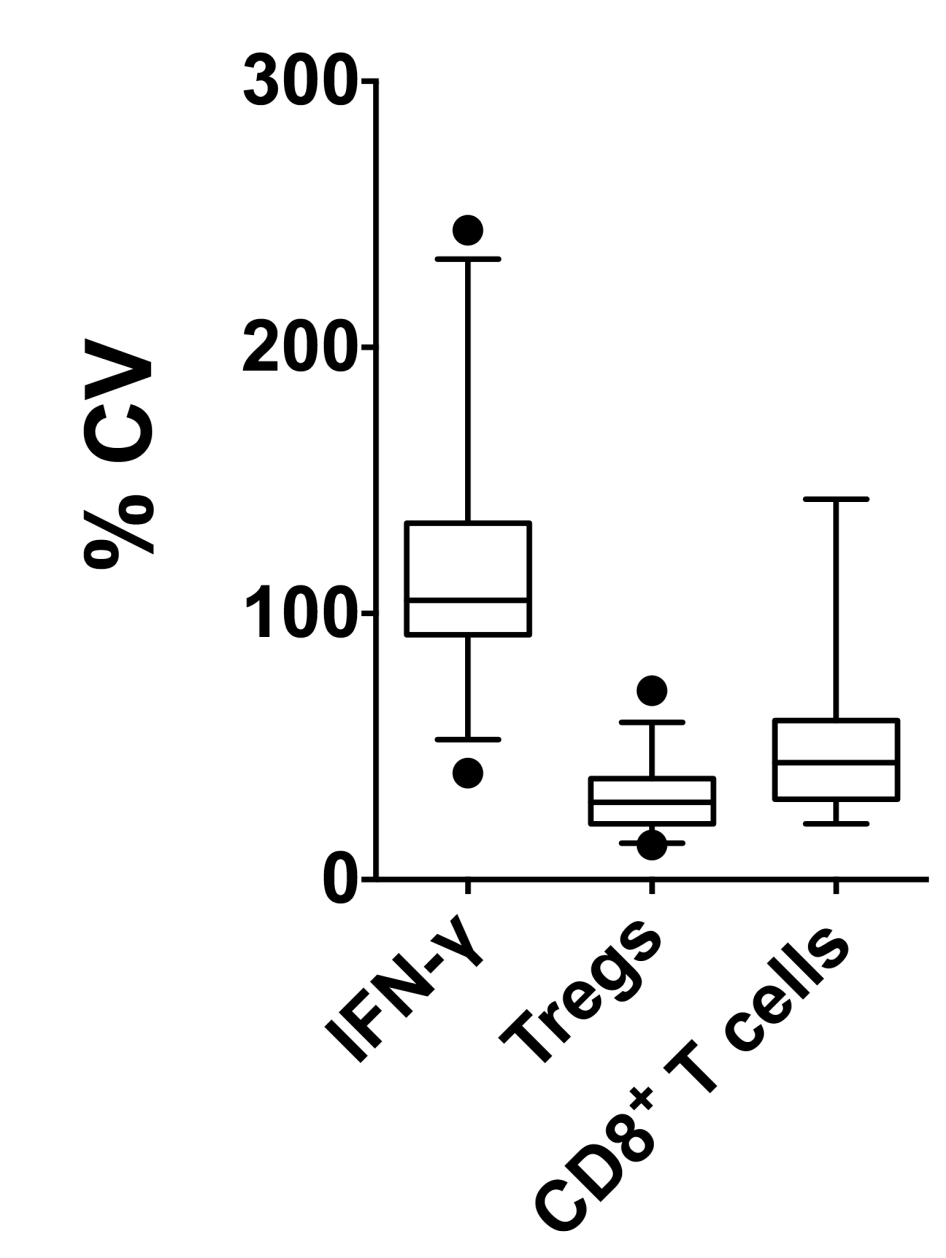
Comparative longitudinal variation (CV) between different parameters within the T1D donor cohort. All antigen-specific (GAD-65, IA-2 and PI) IFN-γ spot counts were pooled for each visit for each donor for all T1D donors, irrespective of DR3- or DR4-positivity (n=33). Similarly, individual frequencies of all diabetes antigen epitope-specific CD8^+^ T-cells were pooled at each visit for each donor but including only the HLA-A2^+^ donors (n=18). For analysis of CD4^+^CD25^+^Foxp3^+^ T-cells, all donors were included in the analysis (n=33). For individual CD4^+^CD25^+^Foxp3^+^ T-cell frequencies or for pooled IFN-γ spot numbers or pooled CD8^+^ T-cell frequencies, the longitudinal variation over all visits was determined as CV. Within the T1D cohort, 5–95% percentile values, the mean, and the 95% CI for CV are shown.

We observed temporal fluctuations over different visits in all of the immunological parameters examined (Figures 1–3). Since the performance of the assays themselves did not show large variations, we concluded that these fluctuations were biological variations within a particular subject. Within the T1D cohort, we observed that production of IFN-γ varied the most with a mean CV of 119.1 ± 8.5% while circulating CD4^+^CD25^+^Foxp3^+^ T-cells showed the least variation with a mean CV of 31.2 ± 2.3%. Finally, detection of antigen-specific CD8^+^ T-cell numbers had a mean CV of 50.4 ± 6.8% (Figure 4 and Table 2). The mean CV for each of these parameters along with the range and 95% CI within the T1D cohort is shown in Table 2.

**Table 2:** Immunological parameters display significant longitudinal variation in T1D donors.

				**95% Confidence Interval**		
	**Minimum CV**	**Maximum CV**	**Mean CV +/- SEM**	**Lower 95% CI of CV**	**Upper 95% CI of CV**	**Relative variation**
**CV (%) in IFN-γ**	40	244	119.1 ± 8.503	101.8	136.5	+++
**CV (%) in CD4^+^CD25^+^Foxp3^+^**	13	71	31.24 ± 2.303	26.55	35.93	+
**CV (%) in absolute Qdot numbers**	21	143	50.44 ± 6.8	36.1	64.79	++

CV in all parameters was quantified as described in Figure 4 and absolute values for minimum, maximum, and mean CV along with 5–95% CI are shown within T1D donors. The number of T1D donors included in each analyses were n=33 for the IFN-γ ELISpot and CD4^+^CD25^+^Foxp3^+^ T-cell analysis and n=18 for HLA-A2-Qdot-multimer analysis.

### Correlation between immunological variation and clinical parameters

**Figure 5:**
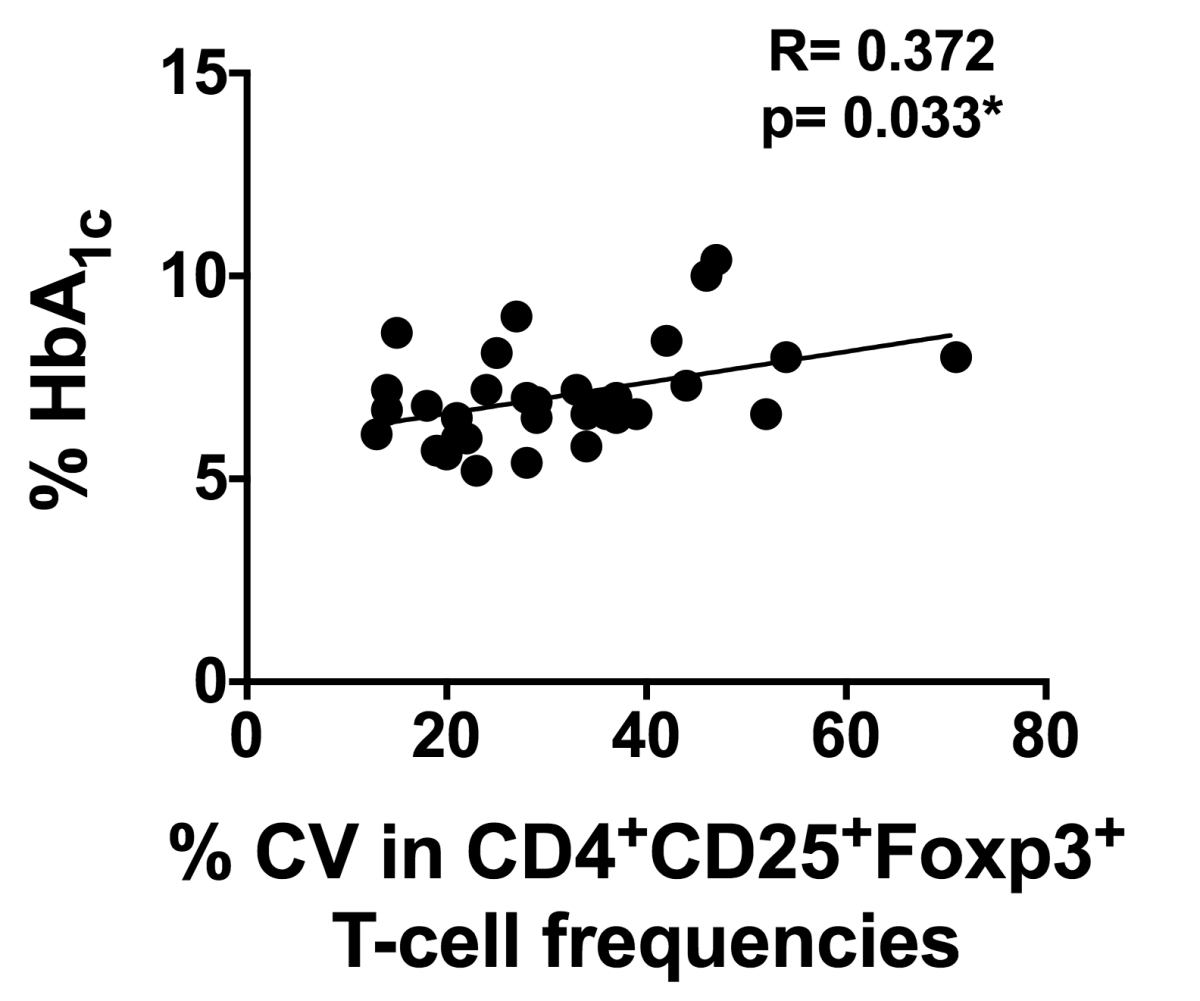
Correlation between immunological variation and clinical parameters. Correlation analysis of variation in CD4^+^CD25^+^Foxp3^+^ T-cell numbers with HbA_1c_ within all T1D subjects (n=33) is shown. Changes in frequencies of CD4^+^CD25^+^Foxp3^+^ T-cells detected between visits were quantitated as CV over all visits. Each dot represents an individual donor.

We then assessed whether the longitudinal variation in T1D donors for any of these parameters correlates with clinical characteristics such as age, disease duration, BMI, or exogenous insulin usage (Figure 5 and data not shown). No correlation was observed between the CV for IFN-γ production or CV for antigen specific CD8^+^ T-cell numbers and any other parameter examined (data not shown). Interestingly, a higher variation in CD4^+^CD25^+^Foxp3^+^ T-cells associated with a higher HbA_1c_ (Figure 5, p= 0.033*) however; the magnitude of the correlation was small (R=0.372). 

### Effect of longitudinal variation on cross-sectional studies

Given the extent of intra-individual temporal variation observed in each of these immunological parameters, assigning a biological effect to an investigational drug in a cross-sectional study with few subjects becomes challenging. Thus, we next determined whether the degree of variation could be reduced by averaging values from multiple visits for each of these parameters. We found that by averaging values from two visits, the CV in these parameters was reduced by ~20%–40% (Table 3). Further, by averaging values from three visits, the CV was reduced by ~30–50%. Thus, averaging values from multiple visits can help offset the measured biological variation. The reduced variation will, in turn, lead to a reduction in the number of subjects that need to be recruited into a particular study to observe a significant effect on an immunological biomarker. Based on the variation seen in our cohort, we calculated that by averaging values from two visits, a treatment effect on these parameters with a 50% effect size could be detected with same power using 1.5–2.2-fold fewer subjects as compared to using values from a single visit. Further, by averaging values from three visits, the number of subjects required to observe similar effects could be reduced by 1.8–3.9-fold (Table 3).

**Table 3:** Power analysis.

	**# visits averaged**	**ELISPOT**	**FACS**	**Qdot**
		**CV IFN-γ**	**CV T_reg_**	**CV CD8^+^**
**Coefficient of variation**	**1**	130%*	31%	51%
	**2**	89%	20%	42%
	**3**	67%	17%	37%
**#donors to detect 50% effect, 90% power**	**1**	194	11	29
	**2**	89	5	20
	**3**	50	4	16
**Fold reduction in #donors versus 1 visit**	**1**	N/A	N/A	N/A
	**2**	2.2	2.2	1.5
	**3**	3.9	2.8	1.8

For each parameter (IFN-γ, Treg and CD8^+^ T-cells), measurements were either taken from individual visits, or averaged over sets of 2 consecutive visits (1-2, 4-5, etc.), or 3 consecutive visits (1-3, 4-6, etc.). Coefficients of variation (CV) were calculated for each donor, and as expected, the mean CV value decreases when averaging measurements over multiple visits. CV values were used to estimate the number of donors required to detect a statistically significant result for a treatment that reduces immune reactivity by 50% with 90% power for each assay. Using measurements averaged over 3 consecutive visits, the number of donors required to detect a significant result can be reduced 1.8 - 3.9 fold. Note that, for the IFN-γ analysis, we included only the 22 donors in the CV calculation for which we had 9 or more datapoints available, to allow comparison with CV values calculated over averages of three visits. Therefore, the CV values for the IFN-γ is slightly different (*130% vs. 119% in Table 2, where all donors were included). For Treg and CD8^+^ T-cell analysis, sufficient datapoints were available for all donors.

## Discussion

Temporal variation in each of these immune parameters was not unexpected [24,25] and could be adversely affected by the minimal frequencies of autoreactive T-cells detectable in the peripheral blood. Thus, quantification of this variation can help us better design future clinical trials. To achieve this goal, here we quantified the intra-individual temporal variation in multiple immunological parameters in our T1D cohort with the CVs ranging from 31% to 119%. Importantly, this variation was not an artifact of non-reproducibility of the assays themselves as intra- and inter-assay CV for each of the assays was low. Rather, it appears that the observed variation witnessed was biological in nature. This finding is of critical importance for using immunological parameters as biomarkers for disease progression and immunological status as well as assessment of immune-based interventions [13,26,27].

In support of our observations, Lachin et al. recently quantified the longitudinal variation in C-peptide production after T1D onset in pediatric patients [28] and determined that this variation could affect the sample size in therapeutic trials. Similarly, Atkinson’s group previously reported [19] longitudinal variation in CD4^+^CD25^+^Foxp3^+^ T-cell numbers (~16% CV) but concluded that this was not highly significant. However, the sample size studied (n = 9) was smaller, and the frequency and the number of blood draws were fewer than in our study; this could account for the difference seen in our study. Further, although not from studies in T1D patients, evidence exists that clinical biomarkers for Alzheimer’s disease [29] also fluctuate within donors who were not undergoing any therapy, supporting our data and the need for evaluation of biological variation.

Surprisingly, the longitudinal variation seen in our T1D subjects was not associated with any clinical feature such as age, duration of disease, BMI, or insulin usage. We did, however, note that a decreased variation in CD4^+^CD25^+^Foxp3^+^ T-cell frequencies from month to month was associated with a lower HbA_1c_ (P = 0.033*). Interestingly, HbA_1c_ did not correlate with variation in either IFN-γ production or in frequencies of CD8^+^ T-cells. Thus the significance of association between HbA_1c_ and variation in CD4^+^CD25^+^Foxp3^+^ T-cell numbers is difficult to ascertain, and further could result from multiple statistical comparisons from a false positive finding. Further, slightly higher but not statistically significant difference in Treg CV in healthy donors, who would presumably have lower HbA_1c_ than T1D donors suggests that this correlation should be interpreted with care. 

Our healthy donor cohort had a near absence of HLA-DR4^+^ and DR3^+^ subjects. For the analysis of variation in IFN-γ production, since we used DR4- or DR3-restricted peptides for antigen stimulation, this could be a potential limitation. However, our results and analyses (both net spot number and SI analysis) of IFN-γ production with the inclusion or exclusion of DR4- or DR3-negative T1D donors did not differ significantly. Thus, while the inclusion of DR4-negative donors could in theory affect the magnitude of responses observed, it is unlikely to significantly change the conclusions drawn from the analysis of longitudinal variation. Moreover, presence of significant HLA-discordant antigen specific responses (both as net spot numbers and SIs) were reported earlier by us as well as others [17,20] in DR4-negative T1D donors arguing against the exclusion of these donors from data analysis. Further, despite the significant differences in ages between the HD and T1D cohort, none of the parameter variation correlated with age nor were there any significant differences between the HD and T1D cohort, providing support to the conclusions drawn from our observations.

There could be a multitude of factors (sampling time, sampling interval, medications in addition to insulin, infections and fevers etc,) that could affect the variation in these immune parameters; most are as yet unknown and could not be controlled for currently. However, some known factors that could potentially have an effect, such as sampling time, and insulin administration (the mode of delivery, route of delivery etc.,) in T1D subjects could be standardized in future studies. Further, plasma glucose concentrations in T1D patients can change rapidly and could affect the variation. But, it would be difficult to control for changes in blood glucose, or to ascertain its effect on the readout as it is unknown how quickly the T-cell repertoire would change in relation to fluctuating blood glucose. We believe that future studies would benefit from determining the identity of factors that affect the variation and controlling for these factors during clinical trials. Our study provides a measure of variation in immune parameters, which was hitherto widely accepted but undetermined and provides a platform from which future studies could benefit.

Our study is the first to provide longitudinal assessment of variation in multiple immunological parameters in the same cohort of patients with T1D. Though the longitudinal variation in the assessed immunological biomarkers was significant, it does not preclude utilizing them as biomarkers in future clinical trials. Based on our findings, we estimate that by averaging data over 3 visits sample size needed to find a significant treatment effect could be reduced by 2- to 4-fold depending on the biomarker. Our results suggest that future trials may require a lead-in phase with multiple blood draws to establish a clear baseline and multiple draws after an intervention should be used to establish an accurate measure of effect. Therefore, a precise understanding of the biological variation of a given biomarker will help determine the appropriate sample size and shape clinical trial design. This will provide a more robust, accurate endpoint for future clinical trials in T1D and aid in the identification of truly efficacious therapies. 

## Supporting Information

Figure S1 **Reproducibility of ELISpot assay.** **A**: 2 × 10^6^ PBMCs from a single HD were stimulated for 48 hrs with CEF viral peptides (positive control) or without (media, negative control). Cells were harvested, adjusted to 3.3 × 10^5^ cells per well and plated in 8-wells of an ELISPOT plate and 16–24 hrs later, IFN-γ spots were developed, numbers of spot-forming cells (SFC) were enumerated and CV (standard deviation ÷ mean) was determined (CV = 7.3%). **B**: ELISPOT assay was performed as in A: but in 4 wells per stimulation, on three separate occasions, using PBMC from a control subject and the CV between the experiments was determined (CV = 13%).
(TIF)

Figure S2 **Reproducibility of Qdot-HLA-A2- multimer assay.** **A**: Viable CD8^+^ T cells were analyzed by gating lymphocytes on FSC-A and SSC-A. Subsequent analysis was performed on single (FSC-W and SSC-H) CD8-Alexa-700 positive live cells (7-AAD negative) after excluding FITC (dump-channel)-positive cells. Cells positive in two specific HLA-A2 multimer channels (e.g., Qdot-585 & -800 for viral epitopes) are selected and cells that are positive in any other channels are excluded using Boolean gating. Cells that are double positive for the two selected Qdots were gated to obtain the frequency of cells that were specific for that epitope. **B**: Frozen PBMCs from a T1D subject from one visit were thawed and stained on two different days to detect the presence of antigen-specific CD8^+^ T-cells against the insulin, IGRP and ppIAPP epitopes. The numbers in the FACS plots indicate the percentage of Qdot-HLA-A2-multimer-positive CD8^+^ T-cells as a fraction of total CD8^+^ T-cells.
(TIF)

Table S1 **Different Qdot-HLA-multimer combinations used to stain PBMCs are shown.**
(DOCX)

Table S2 **Participant information.**
(DOCX)
