# Supplementary figures and images for: Temporal Intra-Individual Variation of Immunological Biomarkers in Type 1 Diabetes Patients: Implications for Future Use in Cross-Sectional Assessment
Source: PLoS One. 2013 Nov 4;8(11):e79383. doi: 10.1371/journal.pone.0079383 (PMC3817042; doi:10.1371/journal.pone.0079383)

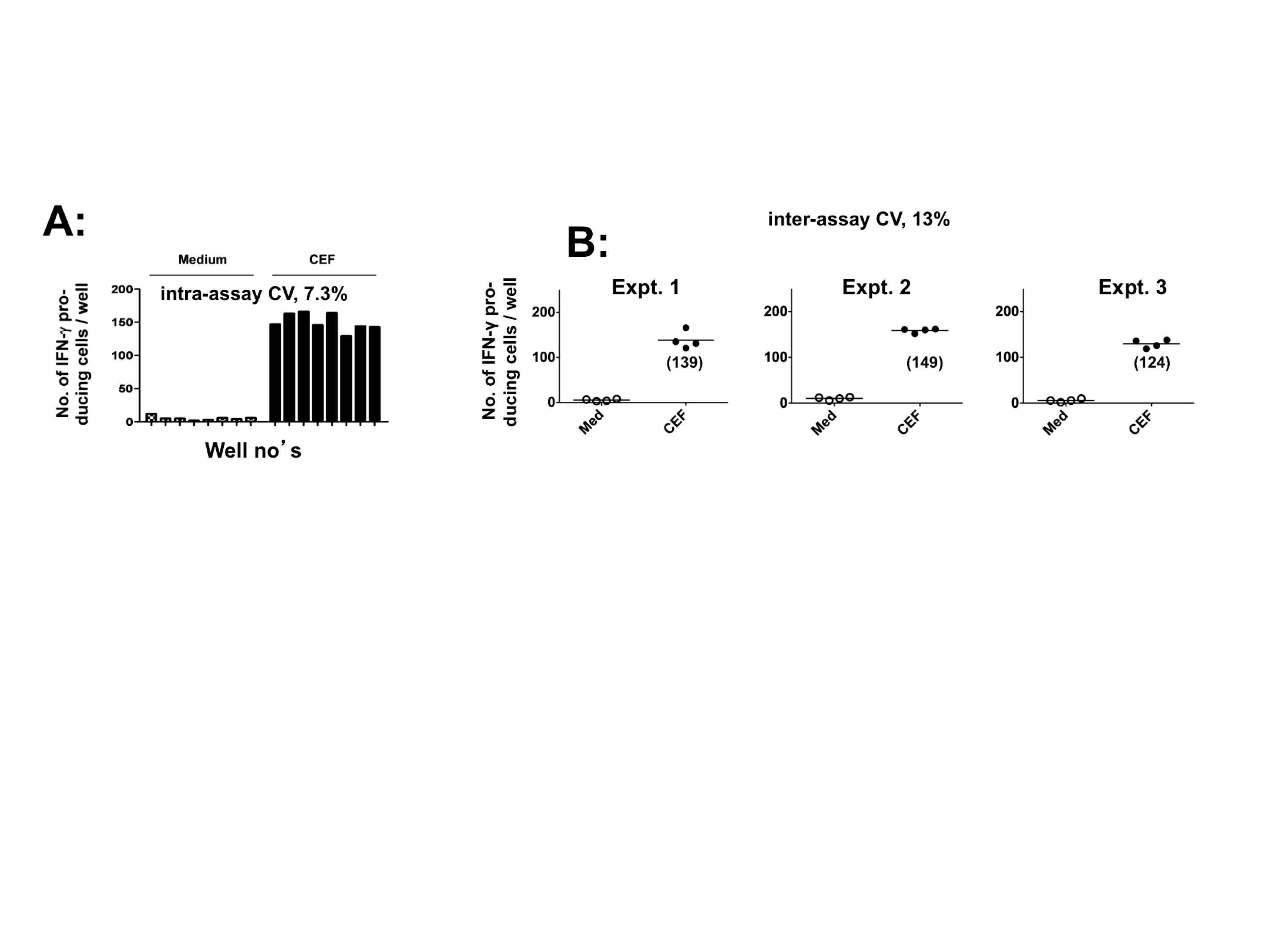

Supplement: Figure S1 — Reproducibility of ELISpot assay. A: 2 × 10^6^ PBMCs from a single HD were stimulated for 48 hrs with CEF viral peptides (positive control) or without (media, negative control). Cells were harvested, adjusted to 3.3 × 10^5^ cells per well and plated in 8-wells of an ELISPOT plate and 16–24 hrs later, IFN-γ spots were developed, numbers of spot-forming cells (SFC) were enumerated and CV (standard deviation ÷ mean) was determined (CV = 7.3%). B: ELISPOT assay was performed as in A: but in 4 wells per stimulation, on three separate occasions, using PBMC from a control subject and the CV between the experiments was determined (CV = 13%). (TIF) [file pone.0079383.s001.tif]

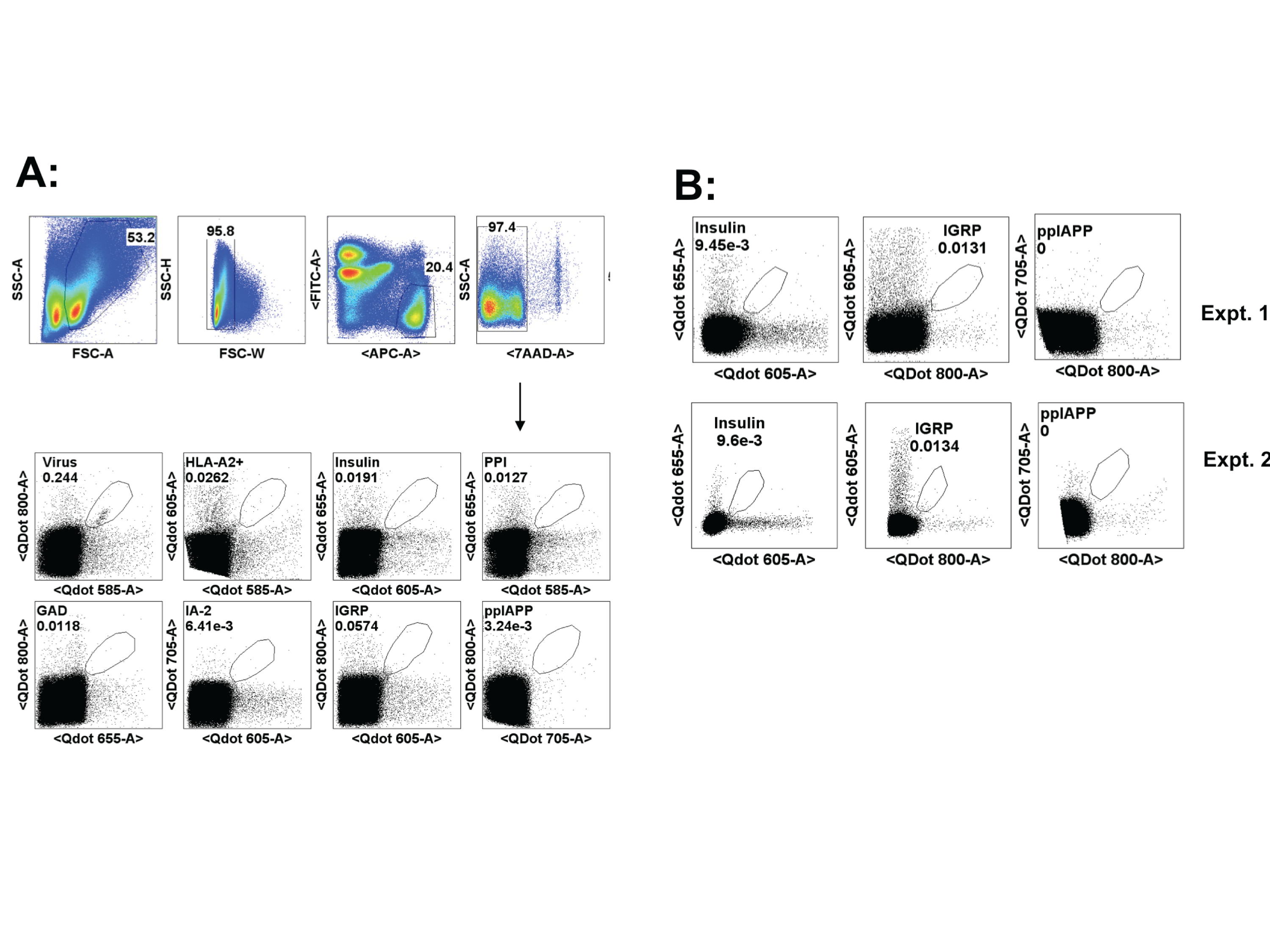

Supplement: Figure S2 — Reproducibility of Qdot-HLA-A2- multimer assay. A: Viable CD8+ T cells were analyzed by gating lymphocytes on FSC-A and SSC-A. Subsequent analysis was performed on single (FSC-W and SSC-H) CD8-Alexa-700 positive live cells (7-AAD negative) after excluding FITC (dump-channel)-positive cells. Cells positive in two specific HLA-A2 multimer channels (e.g., Qdot-585 & -800 for viral epitopes) are selected and cells that are positive in any other channels are excluded using Boolean gating. Cells that are double positive for the two selected Qdots were gated to obtain the frequency of cells that were specific for that epitope. B: Frozen PBMCs from a T1D subject from one visit were thawed and stained on two different days to detect the presence of antigen-specific CD8+ T-cells against the insulin, IGRP and ppIAPP epitopes. The numbers in the FACS plots indicate the percentage of Qdot-HLA-A2-multimer-positive CD8+ T-cells as a fraction of total CD8+ T-cells. (TIF) [file pone.0079383.s002.tif]
