# Supplementary material for: Temporal Intra-Individual Variation of Immunological Biomarkers in Type 1 Diabetes Patients: Implications for Future Use in Cross-Sectional Assessment
Source: PLoS One. 2013 Nov 4;8(11):e79383. doi: 10.1371/journal.pone.0079383 (PMC3817042; doi:10.1371/journal.pone.0079383)
Supplement: Table S1 — Different Qdot-HLA-multimer combinations used to stain PBMCs are shown. (DOCX) [file pone.0079383.s003.docx]

## Table S1: Different Qdot-HLA-multimer combinations used to stain PBMCs are shown.

| **585** | **605** | **655** | **705** | **800** |  |
| --- | --- | --- | --- | --- | --- |
| * |  |  |  | ***** | CMV |
| * |  |  |  | ***** | LMP2 |
| * |  |  |  | ***** | Measles |
| * | * |  |  |  | HLA-A2 |
|  | ***** | ***** |  |  | B10-18 |
| ***** |  | * |  |  | PPI |
|  |  | * |  | * | GAD65 |
|  | * |  | * |  | IA-2 |
|  | * |  |  | * | IGRP |
|  |  |  | ***** | ***** | ppIAPP |

Each HLA-peptide monomer was labeled with two different Qdots with each monomer being labeled with a unique combination of Qdots (except for the viral epitopes).
