# Supplementary material for: Temporal Intra-Individual Variation of Immunological Biomarkers in Type 1 Diabetes Patients: Implications for Future Use in Cross-Sectional Assessment
Source: PLoS One. 2013 Nov 4;8(11):e79383. doi: 10.1371/journal.pone.0079383 (PMC3817042; doi:10.1371/journal.pone.0079383)
Supplement: Table S2 — Participant information. (DOCX) [file pone.0079383.s004.docx]

## Table S2: Participant information.

| **No** | **Age** | **Sex** | **Insulin U/Kg** | **HbA1c** | **GAD65 Aab** | **IA-2 Aab** | **ZnT8 Aab** | **HLA-A2** | **DRB1** | **DRB1** |
| --- | --- | --- | --- | --- | --- | --- | --- | --- | --- | --- |
| 1 | 44 | F | 0.53 | 7 | 1 | 0 | 0 |  | DRB1*03:01 | DRB1*04:05 |
| 2 | 30 | F | 0.64 | 7.2 | 0 | 0 | 1 | A*02:01 | DRB1*01:01 | DRB1*03:01 |
| 3 | 34 | M | 0.54 | 6.6 | 1 | 0 | 0 | A*02:01 | DRB1*07:01 | DRB1*13:03 |
| 4 | 71 | M | 0.51 | 8 | 1 | 1 | 0 | A*02:01 | DRB1*04:01 | DRB1*15:01 |
| 5 | 51 | M | 0.45 | 6.6 | 1 | 1 | 1 |  | DRB1*03:01 | DRB1*04:05 |
| 6 | 26 | M |  | 5.4 | 0 | 0 | 0 | A*02:01 | DRB1*04 | DRB1*07:01 |
| 7 | 30 | M | 0.39 | 5.8 | 1 | 1 | 0 |  |  |  |
| 8 | 47 | M | 0.21 | 5.2 | 1 | 0 | 0 | A*02:01 | DRB1*03:01 | DRB1*03:01 |
| 9 | 49 | F | 0.79 | 9 |  |  |  |  | DRB1*01:03 | DRB1*04 |
| 10 | 34 | M | 0.27 | 5.6 | 1 | 1 | 0 |  | DRB1*04:01 | DRB1*13:02 |
| 11 | 52 | M | 0.39 | 8.4 | 0 | 1 | 0 | A*02:01 | DRB1*03:01 | DRB1*04:01 |
| 12 | 22 | F | 0.10 | 6.8 | 1 | 1 | 1 |  | DRB1*04:01 | DRB1*04:01 |
| 13 | 53 | F | 0.45 | 6 | 0 | 0 | 0 |  | DRB1*13:01 | DRB1*12:01 |
| 14 | 29 | M | 0.88 | 6.1 | 0 | 1 | 0 | A*02:01 | DRB1*03:01 | DRB1*03:01 |
| 15 | 55 | F | 0.47 | 6.5 | 1 | 0 | 0 |  |  |  |
| 16 | 64 | M | 0.47 | 6.8 | 0 | 1 | 0 | A*02:01 | DRB1*04:02 | DRB1*04 |
| 17 | 27 | F | 0.61 | 6 | 1 | 1 | 0 | A*02:01 | DRB1*03:01 | DRB1*04:01 |
| 18 | 77 | M | 0.42 | 6.6 | 0 | 0 | 0 | A*02:01 | DRB1*04 | DRB1*01 |
| 19 | 43 | F | 0.97 | 10.4 |  |  |  | A*02:01 | DRB1*01:01 | DRB1*04:01 |
| 20 | 23 | F | 0.66 | 6.6 | 1 | 0 | 0 |  |  |  |
| 21 | 24 | M | 0.85 | 7 | 0 | 1 | 0 |  | DRB1*04:01 | DRB1*14:04 |
| 22 | 50 | F | 1.15 | 8 |  |  |  |  | DRB1*04:05 | DRB1*15:02 |
| 23 | 30 | F | 0.49 | 10 | 0 | 1 | 0 |  | DRB1*04:01 | DRB1*12:01 |
| 24 | 61 | F |  | 8.1 | 1 | 0 | 0 | A*02:01 | DRB1*07:01 | DRB1*07:01 |
| 25 | 48 | M | 0.47 | 6.9 | 0 | 0 | 0 |  | DRB1*03:01 | DRB1*04:01 |
| 26 | 63 | F | 0.45 | 6.5 | 1 | 1 | 1 | A*02:01 | DRB1*03:01 | DRB1*12:01 |
| 27 | 58 | M | 0.67 | 8.6 | 1 | 0 | 0 |  | DRB1*03:01 | DRB1*04:01 |
| 28 | 60 | F | 0.40 | 7.2 | 1 | 1 | 1 |  | DRB1*04:01 | DRB1*04:01 |
| 29 | 57 | M | 0.29 | 7.3 | 1 | 1 | 0 |  |  |  |
| 30 | 48 | F | 0.39 | 7.2 | 0 | 1 | 0 | A*02:01 | DRB1*07:01 | DRB1*12:01 |
| 31 | 75 | F | 0.37 | 6.7 | 1 | 0 | 0 |  | DRB1*03 | DRB1*13:02 |
| 32 | 28 | F | 0.43 | 6.5 | 0 | 0 | 0 |  | DRB1*01:01 | DRB1*04 |
| 33 | 57 | F | 0.57 | 5.7 | 0 | 0 | 0 |  | DRB1*01 | DRB1*04 |
| *1* | *27* | *F* |  |  | *0* | *0* | *0* | *A*02:01* | *DRB1*03:01* | *DRB1*15* |
| *2* | *35* | *F* |  |  | *0* | *0* | *0* |  |  |  |
| *3* | *32* | *M* |  |  | *0* | *0* | *0* |  | *DRB1*13:01* | *DRB1*13:01* |
| *4* | *28* | *F* |  |  | *0* | *0* | *0* | *A*02:01* | *DRB1*08:11* | *DRB1*11:01* |
| *5* | *35* | *F* |  |  | *0* | *0* | *0* |  |  |  |
| *6* | *41* | *F* |  |  | *0* | *0* | *0* |  |  |  |
| *7* | *35* | *F* |  |  | *0* | *0* | *0* |  | *DRB1*08* | *DRB1*15* |
| 8 | 28 | F |  |  | 0 | 0 | 0 |  | DRB1*12 | DRB1*15 |
| *9* | *23* | *F* |  |  | *0* | *0* | *0* |  | *DRB1*04:03* | *DRB1*12:01* |
| *10* | *29* | *F* |  |  | *0* | *0* | *0* | *A*02:01* | *DRB1*08* | *DRB1*15* |

Participant characteristics including date of diagnosis, disease duration, HbA_1c_, autoantibody status, and HLA-DRB1* status and HLA-A2* status of all donors is shown. Donor numbers 1–33 represent the T1D cohort while italicized donor numbers 1–10 represent the healthy controls.
